# A Major Role of the RecFOR Pathway in DNA Double-Strand-Break Repair through ESDSA in *Deinococcus radiodurans*

**DOI:** 10.1371/journal.pgen.1000774

**Published:** 2010-01-15

**Authors:** Esma Bentchikou, Pascale Servant, Geneviève Coste, Suzanne Sommer

**Affiliations:** Université Paris-Sud 11, CNRS UMR 8621, LRC CEA 42V, Institut de Génétique et Microbiologie, Orsay, France; Baylor College of Medicine, United States of America

## Abstract

In *Deinococcus radiodurans*, the extreme resistance to DNA–shattering treatments such as ionizing radiation or desiccation is correlated with its ability to reconstruct a functional genome from hundreds of chromosomal fragments. The rapid reconstitution of an intact genome is thought to occur through an extended synthesis-dependent strand annealing process (ESDSA) followed by DNA recombination. Here, we investigated the role of key components of the RecF pathway in ESDSA in this organism naturally devoid of RecB and RecC proteins. We demonstrate that inactivation of RecJ exonuclease results in cell lethality, indicating that this protein plays a key role in genome maintenance. Cells devoid of RecF, RecO, or RecR proteins also display greatly impaired growth and an important lethal sectoring as bacteria devoid of RecA protein. Other aspects of the phenotype of *recFOR* knock-out mutants paralleled that of a Δ*recA* mutant: Δ*recFOR* mutants are extremely radiosensitive and show a slow assembly of radiation-induced chromosomal fragments, not accompanied by DNA synthesis, and reduced DNA degradation. Cells devoid of RecQ, the major helicase implicated in repair through the RecF pathway in *E. coli*, are resistant to γ-irradiation and have a wild-type DNA repair capacity as also shown for cells devoid of the RecD helicase; in contrast, Δ*uvrD* mutants show a markedly decreased radioresistance, an increased latent period in the kinetics of DNA double-strand-break repair, and a slow rate of fragment assembly correlated with a slow rate of DNA synthesis. Combining RecQ or RecD deficiency with UvrD deficiency did not significantly accentuate the phenotype of Δ*uvrD* mutants. In conclusion, RecFOR proteins are essential for DNA double-strand-break repair through ESDSA whereas RecJ protein is essential for cell viability and UvrD helicase might be involved in the processing of double stranded DNA ends and/or in the DNA synthesis step of ESDSA.

## Author Summary

*Deinococcus radiodurans* bacterium is among the best-known organisms found to resist extremely high exposures to desiccation and ionizing radiation, both causing extensive DNA double-strand breaks. Because a single unrepaired DNA double-strand break is usually lethal, DNA double-strand breaks are considered as the most severe form of genomic damage. The extreme radioresistance of *D. radiodurans* is linked to its ability to reconstruct a functional genome from hundreds of chromosomal fragments. Genome reconstitution occurs through a two step process: (i) an extended synthesis-dependent strand-annealing process (ESDSA) that assembles genomic fragments in long linear intermediates that are then (ii) processed through recombination to generate circular chromosomes. Here, we demonstrate the essential role of key components of the *D. radiodurans* RecF pathway in ESDSA. We show that (i) inactivation of only one exonuclease (RecJ) results in cell lethality; (ii) cells devoid of RecF, RecO, or RecR display greatly impaired growth; (iii) RecF, RecO, or RecR proteins are essential for radioresistance through ESDSA; and (iv) UvrD helicase has an unexpected crucial function in DNA double-strand-break repair through ESDSA.

## Introduction

The bacterium *Deinococcus radiodurans* is extremely resistant to treatments such as ionizing radiation and desiccation. This resistance can be correlated with the ability of *D. radiodurans* to reconstruct a functional genome from hundreds of radiation or dessication-induced chromosomal fragments, while the genomes of most organisms are irreversibly shattered under the same conditions. The rapid reconstitution of an intact genome is dependent on extended synthesis-dependent strand annealing (ESDSA) and recombination [1],[2]. It was proposed that, following severe DNA damage, the fragmented DNA end is recessed in a 5′–3′ direction, liberating single stranded 3′ overhangs which, through RecA- and RadA-mediated strand invasion, prime DNA synthesis on overlapping fragments [2]. DNA synthesis is initiated by Pol III and elongated by Pol I or by Pol III and the newly synthesized single-strands anneal to complementary single stranded extensions forming long double stranded DNA intermediates which are assembled into intact circular chromosomes by RecA-mediated homologous recombination [2]. Though the dependence of ESDSA on RecA, Pol I, and Pol III activities is well documented [1],[2], little is known about the cellular factors required for the first steps of this process (i.e. the formation of the single stranded 3′ overhangs which promote RecA/RadA - dependent strand invasion to prime DNA synthesis).

Three enzymatic activities are required for presynaptic processing of double stranded DNA ends in the model bacterium *Escherichia coli*: a helicase, a 5′-3′exonuclease, and a mediator function for efficient RecA filament formation onto ssDNA (see for reviews [3]–[5]). All these activities are carried out by the RecBCD complex (or its functional homolog AddAB) which is the major component for initiation of recombinational repair of DNA double-strand-breaks (DSB) in wild-type cells. However, if RecBCD is inactivated, an alternate pathway, the RecF pathway, promotes recombinational DSB repair [6]–[10] in cells containing mutations in *sbcB* (suppressor of *recBC*), which encodes the 3′-5′ exonuclease I, and in *sbcC* (or *sbcD*) [11]. This pathway comprises the 5′-3′ single-strand DNA exonuclease RecJ, the RecQ helicase and the RecF, RecO and RecR proteins that act together to promote loading of RecA onto single stranded DNA.

Whereas examination of the phylogenetic distribution of RecBCD and AddAB complexes revealed that one or the other complex is present in most sequenced bacteria, *D. radiodurans* is naturally devoid of these two complexes but does encode a RecD homologue [12]. RecD protein was shown to be present in the absence of RecBC not only in *D. radiodurans*, but also in firmicutes and *Streptomyces* [13]. The deinococcal RecD protein is expressed and active as a DNA helicase [14]. Further work is required to assign RecD protein to a specific DNA repair pathway because conflicting data have been published concerning the *in vivo* role of RecD in radioresistance [15]–[16]. *D. radiodurans* possesses homologs of the key components of the RecF pathway: RecJ (DR1126), RecQ (DR1289), RecF (DR1089), RecO (DR0819), and RecR (DR0198) suggesting that the RecF pathway is the main recombinational repair pathway in this organism, as observed in other bacteria that lack RecBCD homologs [13]. *D. radiodurans* also lacks homologs of the SbcB nuclease, an inhibitor of the RecF pathway in *E. coli*. Moreover, it was shown that expression *in trans* of the SbcB protein from *E. coli* renders *D. radiodurans* cells radiation-sensitive [17].

In this paper, we investigate the role of the *D. radiodurans* proteins belonging to the RecF pathway in ESDSA and/or homologous recombination. We demonstrate that RecJ exonuclease is an essential protein for cell viability. We show that the RecF, RecO, RecR proteins as well as the RecA protein are absolutely required for massive DNA synthesis during DSB repair whereas RecQ appears to be substituted by the UvrD helicase to play a role in this process. We propose that RecJ, in conjunction with UvrD, could generate the single stranded tails on which RecFOR will stimulate RecA loading. Interestingly, an intact genome could be slowly reconstituted in the absence of RecA, RecF, RecO or RecR, suggesting alternate DSB repair through non-homologous end joining (NHEJ) and/or single-strand annealing (SSA).

## Results

### *recJ* is an essential gene in *D. radiodurans*

**Figure 1:**
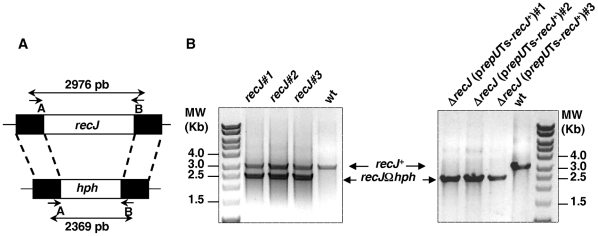
Schematic representation and test of deletion-substitution in the *D. radiodurans recJ* gene. (A) Schematic representation of the allele replacement event in *recJ* gene. Short arrows indicate the position of specific primers used for diagnostic PCR. Primers are described in Table S1. (B) PCR analysis of three independent candidate *recJ* mutants and three independent Δ*recJ* (p*repU*TS-*recJ* ^+^) mutants.

To determine the importance of the RecFOR pathway in DSB repair and radioresistance in *D. radiodurans*, we replaced the coding regions of key genes belonging to this pathway (*recJ*, *recQ*, *recF*, *recO*, and *recR*) with an antibiotic resistance cassette. The deletion-substitution alleles were constructed *in vitro* using the tripartite ligation method [18] and introduced by transformation into *D. radiodurans* to replace the corresponding wild-type alleles via homologous recombination. Because *D. radiodurans* contains from 4 to 10 genome equivalents [19],[20], the transformants were extensively purified on selective media in order to obtain the mutant homogenotes whose purity was verified by PCR. Whereas only few rounds of purification on selective antibiotic plates sufficed to obtain Δ*recQ*, Δ*recF*, Δ*recO* and Δ*recR* homogenotes (see Figure S1), in the case of *recJ*, the wild-type allele was present together with the Δ*recJ* allele even after seven rounds of purification of three independent candidates (Figure 1), suggesting that RecJ protein is essential for cell viability.

**Figure 2:**
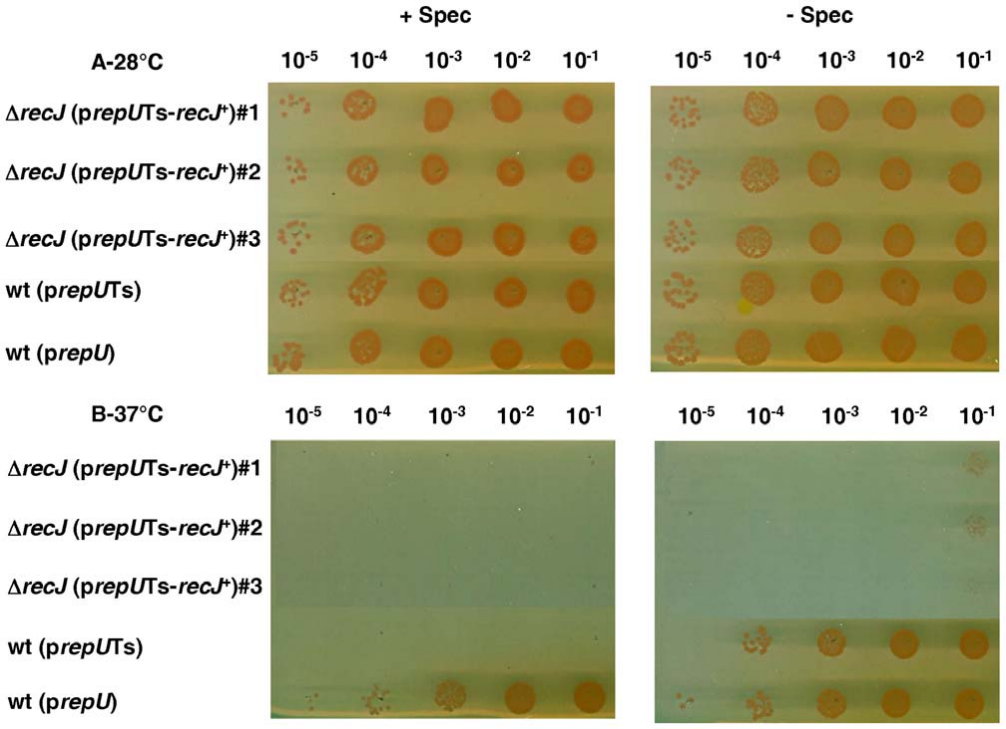
*recJ* is essential for *D. radiodurans* viability. Strains were grown in liquid medium with spectinomycin at 28°C. The dilutions of cells were spotted on medium with or without spectinomycin at 28°C (A) or 37°C (B). Lane 1–3: strain GY14110 [Δ*recJ* (p*repU*Ts-*recJ^+^*)], lane 4: strain GY13781 containing thermosensitive plasmid p13840 (p*repU*Ts), lane 5: strain GY13786 containing non-thermosensitive plasmid p11554 (p*repU*).

To obtain positive evidence for the essentiality of the *recJ* gene, we used the new diagnostic assay described by Nguyen *et al* [21]. For this purpose, the *recJ* gene was cloned onto a p*repU*Ts vector thermosensitive for replication in *D. radiodurans* [21]. The sequence of *DR1126* (*recJ*) in strain ATCC 13939 (GenBank, accession number QG856645) was found to differ from the *DR1126* published sequence [22]. An additional G was found 7 nucleotides upstream the published putative GTG initiation codon and another additional G was found 58 nucleotides before the published putative TGA STOP codon giving rise to a RecJ protein containing 705 aa (versus 684 aa in the RecJ protein predicted from the previously published sequence) with 64 additional amino acids in the N-terminal domain and 43 aa missing in the C-terminal domain of the protein. The predicted sequence of the RecJ protein in strain ATCC 13939 displays a better alignment with the published protein sequences of the *E. coli*, *Deinococcus geothermalis* and *Thermus thermophilus* RecJ proteins (Figure S2). The recombinant plasmid was introduced into a *recJ* ^+^ recipient and the chromosomal copy of *recJ* in the resulting merodiploid strain was inactivated (Figure 1). If *recJ* is an essential gene, the cells will die upon loss of the complementing plasmid at the non permissive temperature. As can be observed in Figure 2 (lanes 1–3), the Δ*recJ* (p*repU*Ts-*recJ* ^+^) bacteria grew normally at 28° (the permissive temperature for the plasmid) but lose viability at 37° (the non-permissive temperature for the plasmid), demonstrating the essentiality of the *recJ* gene.

### Sub-lethal phenotype of Δr*ecF*, Δ*recO*, and Δ*recR* mutants

**Figure 3:**
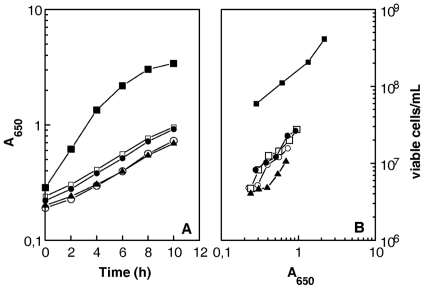
*D. radiodurans* cells devoid of *recA*, *recF*, *recO*, or *recR* genes show reduced plating efficiency. Bacterial strains GY12965 (Δ*recF*, open circles), GY 12966 (Δ*recO*, open squares), GY 12967 (Δ*recR*, filled triangles), GY 12968 (Δ*recA*, filled circles), and wild-type (closed squares) were incubated at 30°C. At different times, incubation samples were taken and the A_650_ values of the cultures and the numbers of viable cells/ml were measured.

The Δ*recF*, Δ*recO*, and Δ*recR* mutants, though viable, showed a greatly impaired growth. Indeed, the mutants had a generation time 4-fold longer than the wild-type (5 hours for the mutants versus 80 min for the wild-type) and comparable to that of a Δ*recA* mutant. Furthermore, cells devoid of RecF, RecO or RecA proteins had a 10-fold reduced plating efficiency as compared to the wild-type strain and this defect was even more pronounced in the Δ*recR* mutant, displaying a 30-fold reduced plating efficiency (Figure 3).

### UvrD helicase is required for rapid repair of DNA double-strand breaks whereas RecQ and RecD helicases are dispensable

In *E. coli*, the RecQ helicase initiates DSB repair via the RecFOR pathway by unwinding duplex DNA in the 3′-5′ direction, while the single stranded DNA exonuclease RecJ hydrolyzes the 5′ strand to provide a DNA-substrate for RecA loading onto the 3′ strand [4],[23].

**Figure 4:**
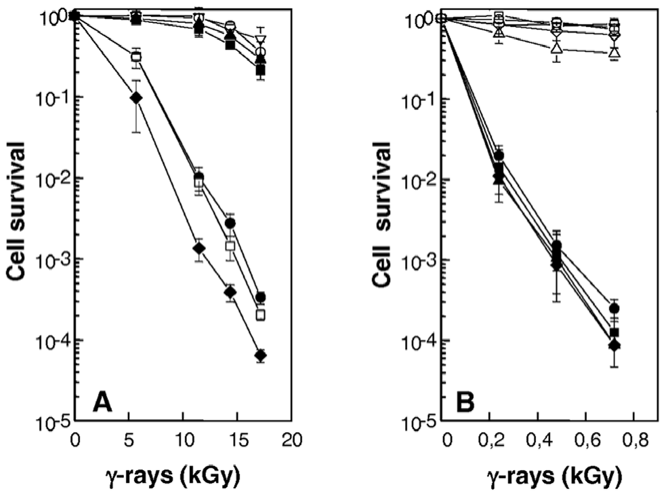
Increased sensitivity to γ-irradiation of cells devoid of RecF, RecO, RecR, or UvrD. (A) Increased sensitivity of cells devoid of *uvrD* gene. R1 (wild-type, open inverted triangles), GY12957 (Δ*recQ*, filled squares), GY12974 (Δ*uvrD*, filled circles), GY12975 (Δ*recQ*Δ*uvrD*, open squares), GY12976 (Δ*recD*Δ*uvrD*, filled diamonds), GY12977 (Δ*recQ*Δ*recD*, filled triangles), GY13130 (Δ*recD*, open circles) bacteria were exposed to γ-irradiation at doses indicated on the abscissa; and cell survival was measured as described in the Materials and Methods. (B) Δ*recFOR* mutants are as sensitive as Δ*recA* mutant to γ-irradiation. Bacterial strains GY12936 (wild-type/p11520, inverted triangles), GY14115 (Δ*recA*/p11559, filled circles), GY14116 (Δ*recO*/p11520, filled diamonds), GY14117 (Δ*recF*/p11520, filled squares), GY14118 (Δ*recR*/p11520, filled triangles), GY14111 (Δ*recA*/p11562: *recA* ^+^, open circles), GY14112 (Δ*recO*/p11860: *recO* ^+^, open diamonds), GY14113 (Δ*recF*/p11862: *recF* ^+^, open squares), GY14114 (Δ*recR*/p11870: *recR* ^+^, open triangles) were exposed to γ-irradiation at doses indicated on the abscissa, and cell survival was measured as described in the Materials and Methods.

We found that inactivation of the RecQ helicase in *D. radiodurans* had no effect on radioresistance, because the knockout mutant was as resistant to γ-irradiation as the wild-type strain (Figure 4A). This result suggests that other helicase(s) might be involved in the initiation step of DSB repair in this organism. We tested the RecD and UvrD helicases for putative roles in DSB repair. We found that a Δ*recD* deletion mutant was as radioresistant as the wild-type strain, whereas a Δ*uvrD* mutant showed a reduction in survival that ranged from 5-fold at 11.6 kGy to more than 100-fold at 17.8 kGy (Figure 4A). However, the mutant still retained significant radioresistance as compared to a repair-deficient Δ*recA* strain (Figure 4B), suggesting that other helicase(s) may overlap in function with UvrD and thus lessen the effect of a Δ*uvrD* mutation. To test this hypothesis, we investigated whether the combined absence of UvrD and RecQ or UvrD and RecD proteins results in a more dramatic effect on radio-resistance. As seen in Figure 4A, the Δ*uvrD* Δ*recQ* double mutant bacteria were not more sensitive to γ-rays than a Δ*uvrD* single mutant. In contrast the Δ*uvrD* Δ*recD* double mutant bacteria were slightly more sensitive to γ-rays than a Δ*uvrD* single mutant, suggesting that the RecD helicase may have a partial back-up function in the absence of UvrD.

**Figure 5:**
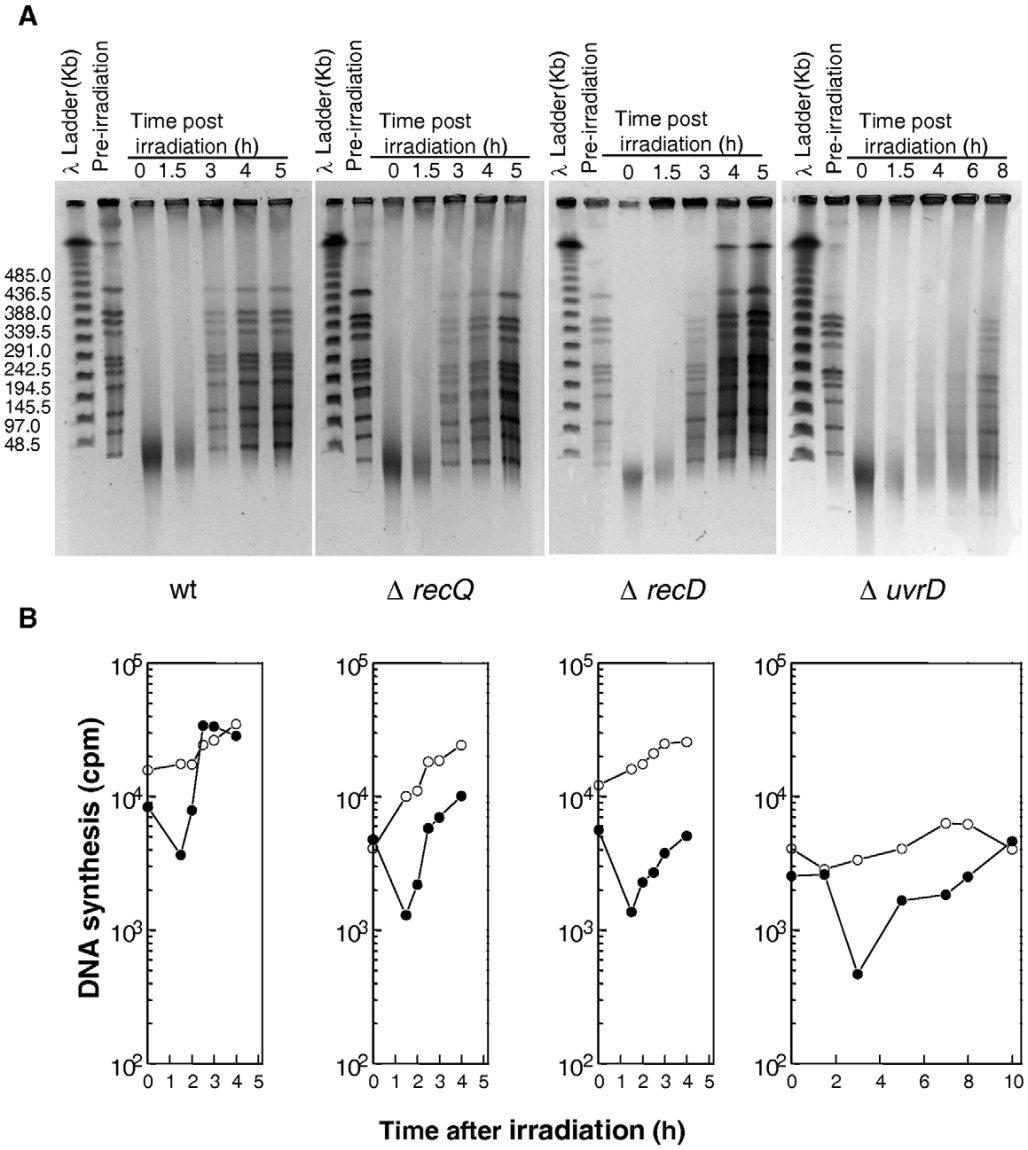
DNA repair and DNA synthesis in Δ*recQ*, Δ*recD*, and Δ*uvrD* mutants. (A) Kinetics of DSB repair in wild-type, Δ*recQ*, Δ*recD*, and Δ*uvrD* mutants followed by pulsed-field gel electrophoresis (PFGE). PFGE shows *NotI* treated DNA from unirradiated cells (lane pre-irradiation) and from irradiated cells (6,8 kGy) immediately after irradiation (0) and at the indicated incubation times (hours). (B) Rate of DNA synthesis in wild-type, Δ*recQ*, Δ*recD*, and Δ*uvrD* mutants. Incorporation of [^3^H]thymidine during 15-min pulse labelling measures the global rate of DNA synthesis in 6.8 kGy-irradiated (filled circles) and unirradiated (open circles) bacteria.

To investigate the possible role(s) of the UvrD helicase in DSB repair, we examined whether the Δ*uvrD* mutant was affected in two key steps of the ESDSA pathway: (i) the reassembly of broken DNA fragments and (ii) the associated massive DNA synthesis. Cells were exposed to 6.8 kGy γ-irradiation, a dose that introduces approximately 200 DSB per genome equivalent in a *D. radiodurans* cell [24]. Recovery from DNA damage was monitored by the appearance of the complete pattern of the 11 resolvable genomic DNA fragments generated by *Not*I digestion [25] and *de novo* DNA synthesis was measured by labelling DNA with a 15 min ^3^H-TdR pulse at different times post irradiation. As seen in Figure 4, Δ*recQ* and Δ*recD* cells repaired DSB with the same kinetics as the wild-type strain, reconstituting an intact genome within 3 h post-irradiation (Figure 5A). In contrast, in Δ*uvrD* bacteria, this process required approximately 8 h (Figure 5A), the kinetics of DSB repair had an increased latent phase (240 min in the mutant versus 90 min in the wild-type) during which DNA degradation took place and a slower rate of fragment assembly. Moreover, resumption of DNA synthesis was delayed in Δ*uvrD* mutant bacteria and its rate was 2-fold lower than that observed in wild-type bacteria (Figure 5B). These results suggest that UvrD plays a major role in DSB repair through ESDSA.

### A major role of RecFOR in *D. radiodurans* radioresistance

The Δ*recF*, Δ*recO* and Δ*recR* mutants were as radiosensitive as a Δ*recA* mutant (Figure 4B). The radiosensitivity of the Δ*recF* and Δ*recO* mutants was fully complemented by a plasmid expressing RecF or RecO proteins *in trans*, whereas, in the case of the Δ*recR* mutant, bacteria expressing *recR* ^+^ *in trans* only recovered 90% of wild-type survival after γ-irradiation (Figure 4B). Because *recR* belongs to a putative operon, the radiosensitivity of the knock-out mutant may be due in part to a polar effect of our construct on a downstream gene or to a sub- or overoptimal plasmid-based expression of the RecR protein.

**Figure 6:**
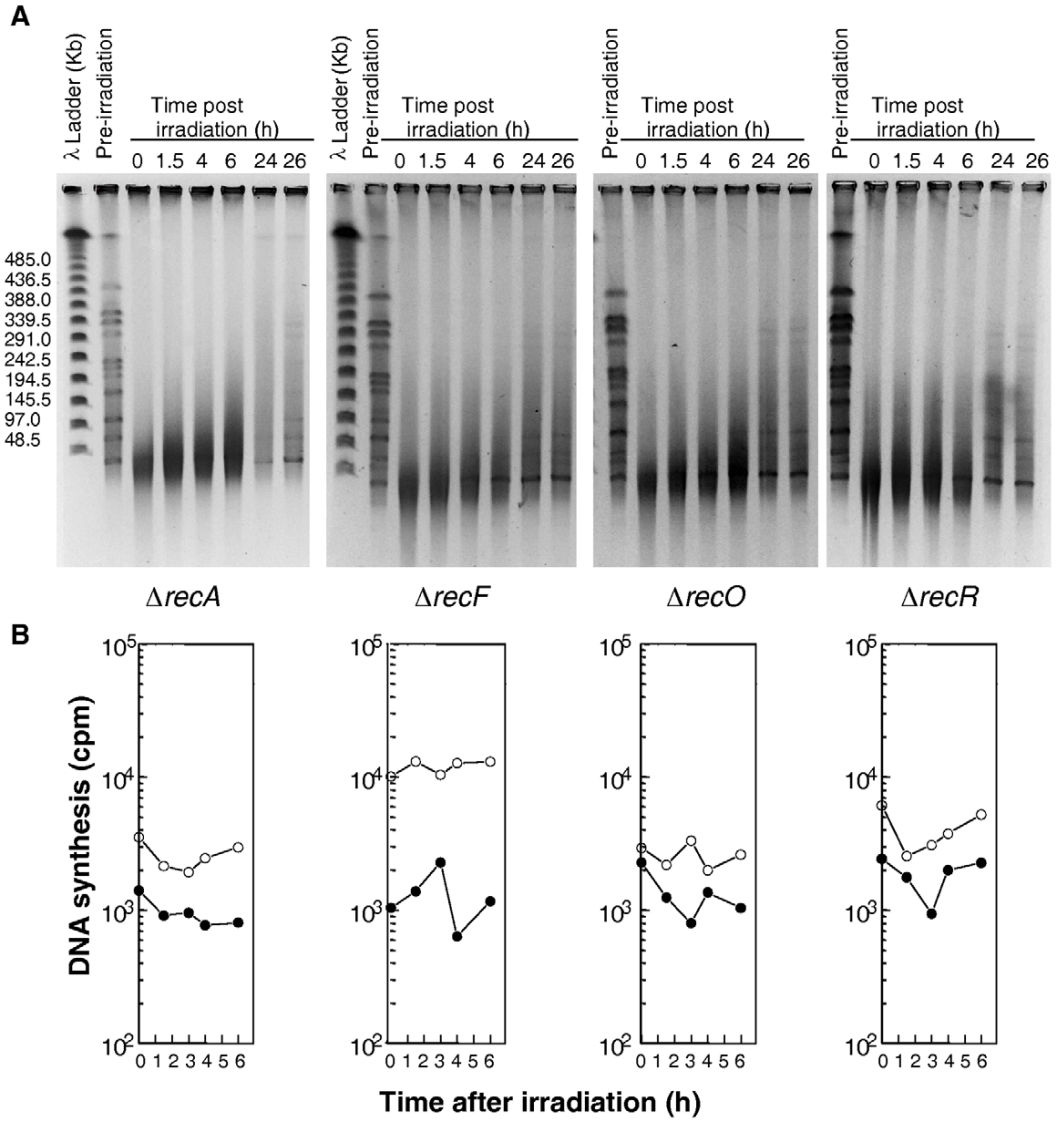
DNA repair and DNA synthesis in Δ*recA*, Δ*recF*, Δ*recO*, and Δ*recR* mutants. (A) Kinetics of DSB repair in wild-type, Δ*recA*, Δ*recF*, Δ*recO*, and Δ*recR* mutants. PFGE shows *NotI* treated DNA from unirradiated cells (lane pre-irradiation) and from irradiated cells (6,8kGy) immediately after irradiation (0) and at the indicated incubation times (hours). (B) Rate of DNA synthesis in wild-type, Δ*recA*, Δ*recF*, Δ*recO*, and Δ*recR* mutants. Incorporation of [^3^H]thymidine during 15-min pulse labelling measures the global rate of DNA synthesis in irradiated (filled circles) and unirradiated (circles) bacteria.

The kinetics of DNA double-strand-break repair in the three mutants was very similar to that observed in a Δ*recA* mutant (Figure 6A). There was a slight and progressive reassembly of the radiation-induced DNA fragments that culminates at 24h post-irradiation incubation in the restitution of a complete pattern of the 11 *Not*I resolvable fragments (Figure 6A). However, only very faint bands of reconstituted chromosome were observed 24h post-irradiation incubation suggesting that a complete genome was only present in a small subpopulation of the mutant cells. The initial degradation of the damaged DNA that can be seen in the wild-type during the first hour of post-irradiation incubation (Figure 5A) was also markedly reduced in the three *recFOR* mutants (Figure 6A), as was previously observed for a Δ*recA* mutant [2]; Figure 6A).

The reconstitution of the complete genomic *Not*I pattern in the irradiated *recFOR* mutants did not result from the multiplication of rare survivors, because there was no observable increase in the number of CFU during 24 hours of incubation of irradiated cells (data not shown). Pulses of ^3^H-TdR showed that no DNA synthesis was observed during the 6 hours following γ-irradiation (Figure 6B) nor during the late fragment assembly in Δ*recF*, Δ*recO* and Δ*recR* bacteria (data not shown), as observed previously in Δ*recA* bacteria [2]. Moreover, the late genome reconstitution in these mutants is not sufficient to ensure cell survival.

In conclusion, our results suggest that RecF, RecO and RecR proteins, like RecA protein, play a central role in Deinococcal radioresistance, probably because they are absolutely required for loading RecA onto its DNA substrate to perform efficient double-strand-break repair via ESDSA and recombinational repair pathways.

## Discussion

Recent studies have shown that the ability of *D. radiodurans* to cope with the DNA-shattering effect of elevated doses of γ-irradiation or dessication involves a robust DNA repair process called extended synthesis-dependent strand annealing (ESDSA) in which long tracts of newly synthesized DNA are made [1],[2]. Whereas the dependence of massive DNA synthesis on Pol I, Pol III and RecA (or its homolog RadA) in ESDSA is well documented [2], little is known about the cellular factors required for the initial steps of this process: (i) the formation of the single stranded 3′ overhangs and (ii) the loading of RecA on this recombinogenic substrate to prime DNA synthesis.

### Major role of RecFOR proteins in DNA double-strand-break repair in *Deinococcus radiodurans*

*D. radiodurans* is naturally devoid of RecB and RecC proteins but contains homologs of key proteins of the *E. coli* RecF pathway: RecJ, RecQ, RecF, RecO and RecR. We found that cells devoid of RecF, RecO or RecR proteins were as radiosensitive as cells devoid of RecA. The Δ*recF*, Δ*recO* and Δ*recR* mutants, as previously shown for a Δ*recA* mutant [2], supported a slow and progressive reassembly of the radiation-induced DNA fragments. As in Δ*recA* cells, genome reassembly was not accompanied by significant DNA synthesis, suggesting that cells devoid of RecF, RecO or RecR proteins are deficient for ESDSA, with repair of DSB probably mediated by RecA-independent pathways, such as single-strand annealing (SSA) or non-homologous end joining (NHEJ). The mutants also showed an important lethal sectoring during normal growth, similar to that observed in a Δ*recA* mutant, in which about 90% of the visible cells failed to give rise to colonies [26]. The similarity of the Δ*recFOR* and Δ*recA* phenotypes supports the hypothesis that RecA activity in *D. radiodurans* is totally dependent on a functional RecF pathway.

Following exposure of *D. radiodurans* to ionizing radiation, there is a rapid and extensive degradation of chromosomal DNA that plays an important role in the repair process in this species (reviewed by [24]). The initial degradation of damaged DNA can be observed using pulsed-field electrophoresis as a reduction of the amount of the double stranded DNA fragments during the first 90 min of post-irradiation incubation in the wild-type cells, prior to the onset of fragment assembly. Slade *et al* observed that DNA degradation is markedly reduced in a Δ*recA* mutant, leading the authors to propose that RecA itself regulates maturation of double-strand ends by controlling both DNA degradation and DNA synthesis [2]. We found that DNA degradation was also reduced in Δ*recF*, Δ*recO* or Δ*recR* mutants, as well as in a Δ*recA* mutant. RecA may play a regulatory role in the control of expression of nuclease-like activities in response to DNA damage, while RecFOR proteins may be indirectly involved in DNA degradation by facilitating the formation of the RecA filament on single stranded DNA. It would be interesting to analyse DNA degradation in the Deinococcal *recA424* mutant, which retains the RecA coprotease activity while remaining deficient in recombination activity [27].

Biochemical studies using RecFOR proteins from *E. coli* indicate that these proteins act together as mediators of the formation of the pre-synaptic RecA filament onto single stranded DNA. Current models agree on the formation of two complexes, RecFR and RecOR. RecOR is generally thought to be responsible for rendering SSB-coated ssDNA accessible to RecA. RecFR targets dsDNA or dsDNA-ssDNA junctions and is responsible for the targeting of RecA to the ssDNA region of gaps [28]–[31]. More recently, it was proposed that RecR is the key component with which RecA interacts, whereas the RecO protein can displace SSB and bind to single stranded DNA independently of RecR, yet does not load RecA until RecR is added [32],[33]. When RecF is present, a RecFOR loading pathway, independent of RecO-SSB interactions, is preferred [33].

Recent X-ray structural analysis of RecO and RecR proteins from *D. radiodurans* confirms the existence of a RecOR complex in this organism. RecR molecules form a ring structure that can encircle both dsDNA and ssDNA [34],[35]. The structure of the RecF protein from *D. radiodurans* has also recently been elucidated, showing that the RecF protein exhibits extensive structural similarity with the head domain of the eukaryotic Rad50 protein [36]. More recently, a model of recognition of the ds-DNA ss-DNA junction in *D. radiodurans* through a DNA-protein and protein-protein interaction was proposed: RecR interacts with ssDNA coated by RecO-SSB, which leads to the elevation of the local concentration of RecR and stimulates RecF binding in the adjacent ds-DNA [37].

### Essentiality of RecJ protein

While inactivation of RecA or RecFOR proteins in *D. radiodurans* reduced cell viability, inactivation of RecJ resulted in a fully lethal phenotype. In other bacterial species, mutations in *recJ* are highly synergistic with those in *recBCD*. In *E. coli*, *recBC recJ* mutants are recombination deficient, extremely UV-sensitive and highly growth disrupted [11],[38]. In *Salmonella typhimurium*, *recB recJ* mutants also display a similar phenotype [39]. More recently, it was shown that a *recJ* knock-out is colethal with *recBCD* or *recD* deletions in *Acinetobacter baylyi* [40]. The strongly reduced viability (or lethality) of *recBC recJ* bacteria was attributed to severe deficiencies in repair of spontaneous DNA damage and inactivated replication forks [39],[40]. It should be noted that, whereas *E. coli* and *S. typhimurium* contain at least three 5′-3′ exonucleases [RecJ, Exo V (RecBCD), Exo VII (XseAB)], the genome of *A. baylyi* encodes only Exo V and RecJ, and that of *D. radiodurans* encodes only RecJ and one of the two subunits of Exo VII. We propose that Exo VII has some back-up activity in *E. coli* or *S. typhimurium* when RecJ and ExoV are inactivated, an activity that is missing in *A. baylyi* and *D. radiodurans*.

In *E. coli*, RecJ and RecFOR were proposed to be required to restore DNA synthesis after UV-induced damage [41],[42]. The mechanism by which lesion-blocked replication forks recover in *E. coli* is thought to involve the formation of reverse replication fork intermediate stabilized by RecA and RecF and degraded by the RecQ-RecJ helicase-nuclease when RecA or RecF are absent [41]. The fork regression allows DNA repair enzymes to remove the blocking lesion, thus restoring processive replication. In the absence of RecJ, the recovery of replication is significantly delayed and both replication recovery and cell survival become dependent on translesion synthesis by DNA polymerase V [42]. *D. radiodurans* does not encode a bypass DNA polymerase belonging to the Y family, and under these conditions RecJ may be essential for restoration of replication forks after arrest, even in cells not treated by DNA damaging agents. Frequent DNA double-strand-breaks were thought to arise spontaneously ranging from 0.2–1 per genome replication in *E. coli* [4],[43]. However, a more direct quantification of DNA double-strand-breaks indicated that the rate of spontaneous breakage is 20 to 100-fold lower than predicted, only one percent of the cells having one or more DNA double-strand-breaks per genome replication [44].

Because cells devoid of RecA or RecFOR are viable, the Δ*recJ* lethal phenotype cannot be only due to a possible deficiency in DSB repair, leading us to postulate that RecJ is required *in D. radiodurans* for more than one cellular process and that inactivation of all of these processes (DSB repair, fork reversion, restoration of a fork structure after regression …) may be lethal for the cell.

### Involvement of UvrD in DNA double-strand-break repair

In *E. coli*, the RecJ exonuclease has been mainly associated with the RecQ helicase in recombination and repair (see, for review, [4]). The RecQ protein from *D. radiodurans* shows unusual domain architecture with three tandem HRDC (Helicase RNase D C-terminal) domains in addition to the conserved helicase and RQC (RecQ C-terminal) domains. The tandem arrangement of HRDC domains regulates the specificity of the binding of RecQ to DNA substrates [45],[46]. Here, we found that Δ*recQ* mutants displayed a wild-type level of resistance to γ-irradiation, exhibiting the same kinetics as the wild-type strain for fragment reassembly and DNA synthesis after irradiation. In another report, a *recQ* knock-out mutant was shown to be highly sensitive to H_2_O_2_ and slightly more sensitive than the wild-type strain to elevated γ-irradiation doses [46]. It was recently proposed that *recQ* deletion, by causing transcriptome alteration, would generate ROS accumulation and Fe and Mn alterations [47]. Our findings suggest that the RecQ helicase in *D. radiodurans* plays only a minor role in DSB repair, probably as consequence of redundant functions provided by other helicase(s). Mutants devoid of RecD behave like Δ*recQ* mutants in that they show wild-type radioresistance and repair capacity. The Deinococcal RecD protein has been characterized *in vitro* as a helicase with 5′-3′ polarity (opposite to that of RecQ) and low processivity [14].

In contrast, we found that inactivation of UvrD (helicase II) markedly reduced Deinococcal radioresistance and severely delayed the kinetics of DSB repair. UvrD has been largely characterized for its role in nucleotide excision repair (NER) and mismatch repair (MMR) in *E. coli* (reviewed by [48]). However, the altered kinetics of repair and the radiosensitivity of Δ*uvrD* bacteria are unlikely to result from a deficiency in the NER pathway because *uvrA* deficient mutant bacteria display a wild-type survival pattern following exposure to ionizing-radiation (Figure S3). The Δ*mutS* bacteria deficient for MMR were also shown to be as radioresistant as wild-type bacteria [18]. Interestingly, the delayed kinetics of DSB repair in cells devoid of UvrD coincided with DNA synthesis (albeit significantly less extensive than that observed in the wild-type cells) suggesting that ESDSA repair could take place but only inefficiently in this mutant. We propose that UvrD is involved in ESDSA and that the redundant activity of other helicase(s) is responsible for the residual DNA repair capacity observed in the Δ*uvrD* mutant. The fact that the Δ*recQ*Δ*uvrD* and the Δ*recD*Δ*uvrD* double mutant bacteria were not as radiosensitive as Δ*recA* bacteria suggests that neither RecQ nor RecD can solely fulfil this role, and that other helicase(s) may be involved. Helicase IV (HelD) has been implicated as partner of the RecJ exonuclease in the RecF pathway in *E. coli*, together with Helicase II and RecQ [49]. Mutational inactivation of Helicase IV has no effect on the radioresistance of *D. radiodurans* [50]. Alternatively, RecA itself, by binding to double stranded DNA ends, could unwind DNA and provide a DNA substrate for RecJ or another 5′-3′ exonuclease. Indeed, *in vitro*, the *D. radiodurans* RecA protein binds preferentially to double stranded DNA [51].

In *E. coli*, the UvrD protein was not shown to be required for DNA double-strand-break repair. In contrast, it was shown to possess an anti-recombination activity, which has been related to its capacity to disrupt the RecA nucleoprotein filament [52],[53]. This activity is conserved among many species [54]. Thus, as in other species, the *D. radiodurans* UvrD protein might not be involved directly in the maturation of DNA double-strand ends. Several observations suggest that *E. coli* UvrD may be involved in DNA replication [55]–[58] and it was shown to be required for DNA replication of several different rolling-circle plasmids in *E. coli* [59]. Thus, the *D. radiodurans* UvrD protein might also act in the DNA synthesis step of ESDSA.

### A scenario for DNA double-strand-break repair through ESDSA in *D. radiodurans*

**Figure 7:**
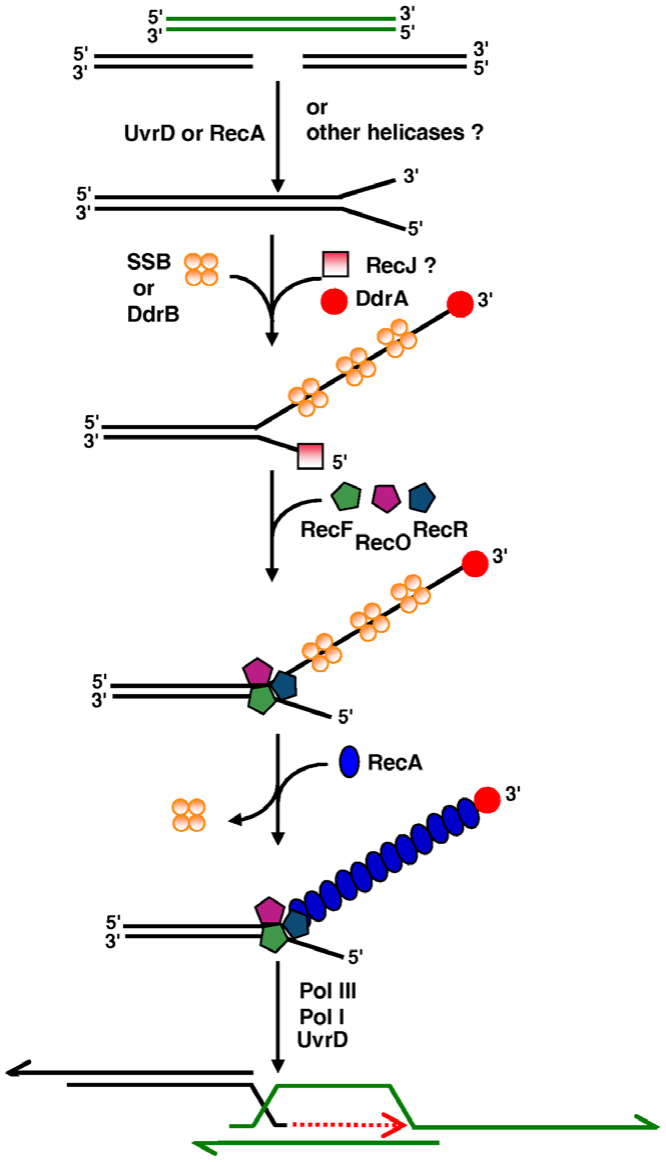
Model of initiation of DNA double-strand-break repair through ESDSA in *D. radiodurans*.

Taking into account our results and those of others [2],[60],[61], we propose a model for the role of the proteins of the RecF pathway in ESDSA (Figure 7). In this model, RecJ or an as-yet unidentified exonuclease associated with the UvrD helicase, could generate 3′ single stranded DNA ends required for priming of massive DNA synthesis. Alternatively, RecA itself, by binding to double stranded DNA ends, could unwind DNA and provide a DNA substrate for RecJ or another exonuclease.

Analysis of the transcriptome of *D. radiodurans* revealed a large group of genes that are up-regulated in response to either desiccation or ionizing radiation [62]. The deinococcal specific *ddrA* (*DR0423*) and *ddrB* (*DR0070*) genes were found among the most highly induced in response to each stress and their inactivation promotes sensitization of the mutant cells to ionizing radiation [62]. The DdrA protein is involved in protection of 3′ DNA single stranded ends [60] and presumably ensures long-lived recombinational substrates [63]. The DdrB protein binds single stranded DNA but not duplex DNA and is the prototype of a new bacterial SSB family [61]. The induction of an alternative SSB following irradiation has potentially broad significance for efficient genome reconstitution. We propose that during initiation of ESDSA, DdrA protects the 3′ DNA ends whereas SSB or the SSB-like DdrB binds to single stranded DNA.

Our results supporting the idea that RecA activity in *D. radiodurans* is totally dependant on a functional RecF pathway, lead us to propose that RecFOR renders SSB or DdrB- coated single stranded DNA accessible to RecA and favors formation of a RecA nucleoprotein filament required for invasion of a double stranded homologous DNA. Finally, as described previously [2], Pol III and Pol I can promote DNA synthesis, eventually with the help of the UvrD helicase.

Moreover, the compact *D. radiodurans* nucleoid structure that remains unaltered after high-dose γ-irradiation may passively contribute to radioresistance by preventing the dispersion of free DNA ends [64],[65]. Such a condensed genome may provide suitable scaffolds for DNA repair through ESDSA, recombinational and/or DNA end joining processes.

In conclusion, we demonstrate the essential role of key components of the *D. radiodurans* RecF pathway in ESDSA. We show for the first time that (i) inactivation of only one exonuclease, RecJ, results in cell lethality (ii) cells devoid of RecF, RecO or RecR display greatly impaired growth (iii) RecF, RecO or RecR proteins are essential for radioresistance through ESDSA (iv) UvrD helicase has an unexpected crucial function in DNA double-strand-break repair through ESDSA.

## Materials and Methods

### Bacterial strains, cultures, media, and transformation

Bacterial strains and plasmids are listed in Table 1 and Table 2, respectively. The *Escherichia coli* strain DH5α was used as the general cloning host and strain SCS110 was used to propagate plasmids prior to introduction into *D. radiodurans* via transformation [66]. All *D. radiodurans* strains were derivatives of strain R1 (ATCC 13939). *D. radiodurans* was grown in TGY2X (1% tryptone, 0.2% dextrose, 0.6% yeast extract) or in TGYA (0.5% tryptone, 0.2% dextrose, 0.15% yeast extract) at 30°C with aeration or on TGY1X plates solidified with 1.5% agar. *E. coli* strains were grown in Luria-Bertani (LB) broth (Gibco Laboratories). When necessary, media were supplemented with the appropriate antibiotics used at the following final concentrations: chloramphenicol 3 µg/mL for *D. radiodurans*; kanamycin 6 µg/mL for *D. radiodurans*; tetracycline 2.5 µg/mL for *D. radiodurans*; hygromycin 50 µg/mL; spectinomycin 40 µg/mL for *E. coli* and 75 µg/mL for *D. radiodurans*. Transformation of *D. radiodurans* with PCR products, genomic DNA, or plasmids was performed as previously described [26].

**Table 1:** Bacterial strains.

Bacterial strains	Genotype	Source or reference
*E. coli*		
**DH5α**	*supE44 ΔlacU(φ80lacZΔM15) hsdR17 recA1 endA1 gyrA96 thi-1 relA1*	Laboratory stock
**SCS110**	*endA dam dcm supE44* Δ(*lac*-*proAB*) (*F’traD36 proAB lacI^q^ZΔM15*)	Laboratory stock
*D. radiodurans*		
**R1**	ATCC 13939	Laboratory stock
**302**	*uvrA1*	[70]
**GY10973**	*amyEΩ*P*tufA:lacI-kan*	[69]
**GY12936**	R1/p11520	[68]
**GY12957**	Δ*recQ*Ω*cat*	this work
**GY12965**	Δ*recF*Ω*cat*	this work
**GY12966**	Δ*recO*Ω*hph*	this work
**GY12967**	Δ*recR*Ω*kan*	this work
**GY12968**	Δ*recA*Ω*kan*	this work
**GY12974**	Δ*uvrD*Ω*hph*	this work
**GY12975**	Δ*recQ*Ω*cat*Δ*uvrD*Ω*hph*	this work
**GY12976**	Δ*recD*Ω*kan*Δ*uvrD*Ω*hph*	this work
**GY12977**	Δ*recQ*Ω*cat*Δ*recD*Ω*kan*	this work
**GY12978**	non homogenotized Δ*recJ*Ω*hph*	this work
**GY13130**	Δ*recD*Ω*kan*	Laboratory stock
**GY13781**	GY10973/p13840	[21]
**GY13786**	GY10973/p11554	[21]
**GY14105**	GY10973/p11869 (p*repU*Ts::*recJ^+^*)	this work
**GY14110**	as GY14105 but Δ*recJ*Ω*hph*	this work
**GY14111**	Δ*recA*Ω*kan*/p11562 (*recA* ^+^)	this work
**GY14112**	Δ*recO*Ω*hph*/p11860 (*recO* ^+^)	this work
**GY14113**	Δ*recF*Ω*cat*/p11862 (*recF* ^+^)	this work
**GY14114**	Δ*recR*Ω*kan*/p11870 (*recR* ^+^)	this work
**GY14115**	Δ*recA*Ω*kan*/p11559	this work
**GY14116**	Δ*recO*Ω*hph*/p11520	this work
**GY14117**	Δ*recF*Ω*cat*/p11520	this work
**GY14118**	Δ*recR*Ω*kan*/p11520	this work

**Table 2:** Plasmids.

Plasmids	Description	Reference
**pGTC101**	Source of chloramphenicol cassette in *D.radiodurans*	[71]
**pKatHPH4**	Source of hygromycin cassette in *D.radiodurans*	gift of I. Narumi
**p11086**	Source of kanamycin cassette in *D.radiodurans*	laboratory stock
**p11520**	Derivative of pI8; Spc^R^ in *D.radiodurans*	[68]
**p11559**	Expression vector; P*_Spac_*,P*tufA*::*lacI*, Spc^R^ in *E.coli* and in *D.radiodurans*	[18]
**p11554**	Shuttle vector *E. coli*/*D. radiodurans*, Spc^R^	[21]
**P11562**	p11559: *recA^+^*	[63]
**P11860**	p11520 with a PCR fragment encoding *recO*	this work
**P11862**	p11520 with a PCR fragment encoding *recF*	this work
**P11870**	p11520 with a PCR fragment encoding *recR*	this work
**p11830**	Vector thermosensitive for replication in *D. radiodurans*, Spc^R^, p*repU*Ts	[21]
**p13840**	p11830 P*_Spac_-term 116*	[21]
**p11869**	p13840: p*repU*Ts::*recJ*	this work

### DNA manipulations

Plasmid DNA was extracted from *E. coli* using the QIAprep spin miniprep kit (Qiagen). Chromosomal DNA of *D. radiodurans* was isolated as previously described [67]. Amplification of plasmid or genomic DNA by PCR was performed with DyNAzyme EXT DNA polymerase (Finnzyme) or Extensor Hi-Fidelity PCR enzyme Mix (ABgene). Oligonucleotides used are listed in Table S1.

### Deletion of genes in *D. radiodurans*

The *recF*, *recO*, *recR*, *recA*, *uvrD*, *recD*, *recQ*, *recJ* disruption mutants were constructed by the tripartite ligation method [18]. The mutated alleles constructed *in vitro* were then used to transform *D. radiodurans* to replace their wild-type counterpart by homologous recombination. The genetic structure and the purity of the mutants were checked by PCR using primers described in Table S1.

### Construction of plasmids

Plasmid p11869 is a derivative of the thermosensitive plasmid p13840 [21]. To construct p11869, the *recJ* gene was amplified by PCR using the primer pair (PS441/PS442) and the product was cloned into plasmid p13840 between the *NdeI*/*XhoI* sites.

Plasmids p11862, p11860 and p11870 carrying the *recF*, *recO*, *recR* genes, respectively, under the control of their natural promoter were used to express the *recF*, *recO*, *recR* genes in a Δ*recF*, Δ*recO*, Δ*recR* background. To construct plasmid p11860, the *recO* gene was amplified by PCR using the primer pair (PS402/PS403) and the resultant product was cloned into plasmid p11520 [68] between the *SacI*/*BamHI* sites. Plasmid p11870, containing the *recR* gene, was constructed in a similar manner using the primer pairs PS414/PS415. The *recF* gene was cloned into plasmid p11520 between the *SpeI*/*BglII* sites in a similar manner using the primers PS410/PS411 to obtain p11862. All constructions were verified by DNA sequencing.

Plasmid p11562 [63], expressing *recA* from a P*_Spac_* promoter, was used to transform GY12968: Δ*recA*Ω*kan* giving rise to strain GY14111. The expression of *recA* was induced by adding 10 mM IPTG to the media.

### Treatment of *D. radiodurans* with γ-irradiation

Exponential cultures, grown in TGY2X (supplemented with spectinomycin when necessary), were concentrated to an A_650_ = 10 in TGY2X and irradiated on ice with a ^137^Cs irradiation system (Institut Curie, Orsay, France) at a dose rate of 44.7 Gy/min. Following irradiation, diluted samples were plated on TGY plates. Colonies were counted after 3–4 days incubation at 30°C.

### Assay of *recJ* gene essentiality

The essentiality of genes was evaluated in a growth experiment in which the strains grown at 28°C in liquid medium with spectinomycin, were serially diluted, plated on TGY agar and incubated at 28°C or 37°C in the presence or absence of spectinomycin [21].

### Kinetics of DNA repair measured by pulse-field gel electrophoresis

Non-irradiated or irradiated (6.8 kGy) cultures were diluted in TGY2X to an A_650_ = 0.2 and incubated at 30°C. At different post-irradiation recovery times, culture aliquots (5mL) were removed to prepare DNA plugs as described previously [60]. The agarose embedded DNA plugs were digested for 16 h at 37°C with 10 units of *NotI* restriction enzyme. After digestion, the plugs were subjected to pulsed field gel electrophoresis as described previously [69].

### Rate of DNA synthesis measured by DNA pulse labelling

The rate of DNA synthesis was measured according to a modified protocol from Zahradka *et al* [1]. Exponential cultures, grown in TGYA, were concentrated to an A_650_ = 20 in TGYA and irradiated as described previously. Non-irradiated or irradiated cultures (6.8 kGy) were diluted in TGYA to an A_650_ = 0.2 and incubated at 30°C. At different time 0.5mL samples were taken and mixed with 0.1mL pre-warmed TGYA containing 4.8 µCi [methyl-^3^H]thymidine (PerkinElmer, specific activity 70–90 Ci/mmol). Radioactive pulses of 15 min were terminated by addition of 2 mL ice-cold 10% TCA. Samples were kept on ice for at least 1 h, and then collected by vacuum filtration onto Whatman GF/C filters followed by washing twice with 5mL 5% TCA and twice with 5mL 96% ethanol. Filters were dried for 10 min under a heat source and placed in 4 mL scintillation liquid. The precipitated counts were measured in a liquid scintillation counter (Packard, TRI- carb 1600 TR).

## Supporting Information

Figure S1 Schematic representation and test of deletion-substitution of *D. radiodurans recF*, *recO*, *recR*, and *recQ* genes. (A) schematic representation of the allele replacement event of *recF* (A.1), *recO* (A.2), *recR* (A.3), and *recQ* (A.4) genes. Short arrows indicate the position of specific primers used for diagnostic PCR. Primers are described in Table S1. (B) PCR analysis of Δ*recF* (B.1), Δ*recO* (B.2), Δ*recR* (B.3), and Δ*recQ* (B.4) mutants.
(0.72 MB TIF)

Figure S2 Alignment of the *D. radiodurans* ATCC13939 RecJ protein. Alignment of the *D. radiodurans* ATCC13939 RecJ protein with its TIGER sequence and the corresponding *Deinococcus geothermalis*, *Termus thermophilus*, and *E. coli* RecJ protein. The alignment was generated using Clone Manager program. Shading was based on amino acid identity (green boxes).
(0.04 MB DOC)

Figure S3 Nucleotide Excision Repair deficient *uvrA* bacteria are as radioresistant as the wild type. R1 (wild type, open inverted triangles), GY12974 (Δ*uvrD*, filled circles), and GY9614 (*uvrA1*, filled squares) bacteria were exposed to γ-irradiation at doses indicated on the abscissa, and cell survival was measured as described in the Materials and Methods.
(0.10 MB TIF)

Table S1 Overview of primers used for construction of mutant strains, cloning, and diagnostic PCR experiments.
(0.04 MB DOC)
