# Supplementary figures and images for: A Major Role of the RecFOR Pathway in DNA Double-Strand-Break Repair through ESDSA in Deinococcus radiodurans
Source: PLoS Genet. 2010 Jan 15;6(1):e1000774. doi: 10.1371/journal.pgen.1000774 (PMC2806897; doi:10.1371/journal.pgen.1000774)

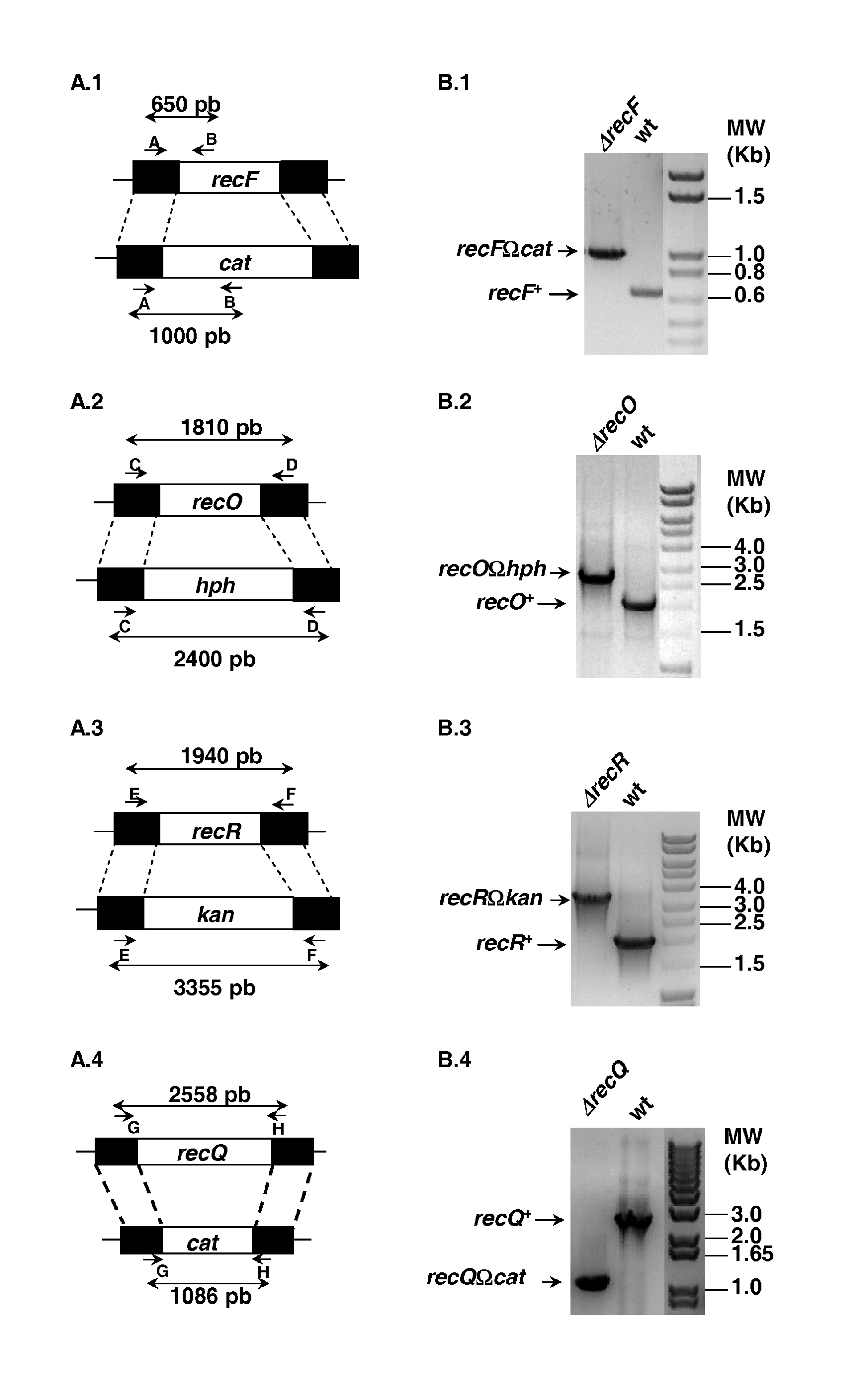

Supplement: Figure S1 — Schematic representation and test of deletion-substitution of D. radiodurans recF, recO, recR, and recQ genes. (A) schematic representation of the allele replacement event of recF (A.1), recO (A.2), recR (A.3), and recQ (A.4) genes. Short arrows indicate the position of specific primers used for diagnostic PCR. Primers are described in Table S1. (B) PCR analysis of ΔrecF (B.1), ΔrecO (B.2), ΔrecR (B.3), and ΔrecQ (B.4) mutants. (0.72 MB TIF) [file pgen.1000774.s001.tif]

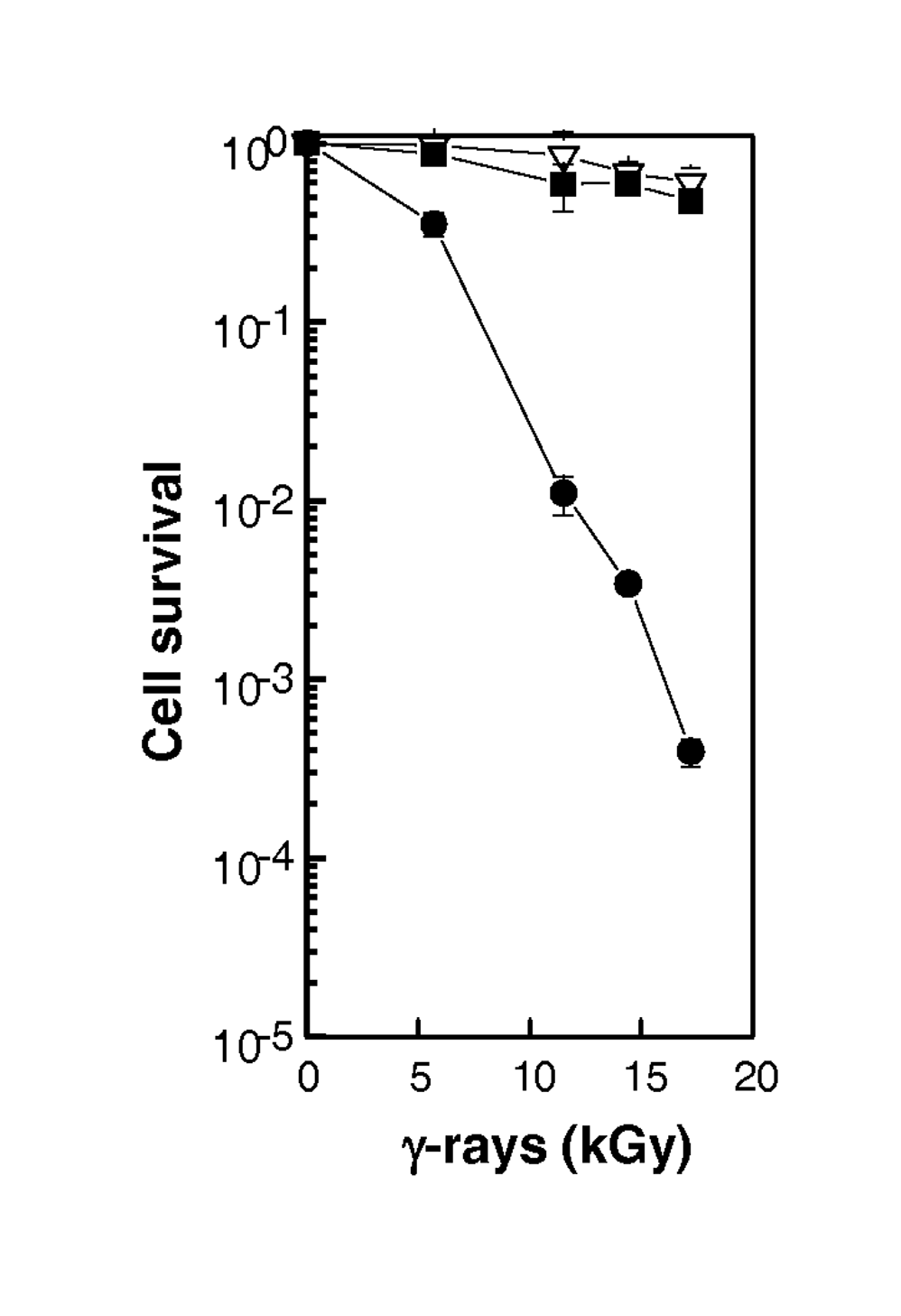

Supplement: Figure S3 — Nucleotide Excision Repair deficient uvrA bacteria are as radioresistant as the wild type. R1 (wild type, open inverted triangles), GY12974 (ΔuvrD, filled circles), and GY9614 (uvrA1, filled squares) bacteria were exposed to γ-irradiation at doses indicated on the abscissa, and cell survival was measured as described in the Materials and Methods. (0.10 MB TIF) [file pgen.1000774.s003.tif]
