# Supplementary material for: A Major Role of the RecFOR Pathway in DNA Double-Strand-Break Repair through ESDSA in Deinococcus radiodurans
Source: PLoS Genet. 2010 Jan 15;6(1):e1000774. doi: 10.1371/journal.pgen.1000774 (PMC2806897; doi:10.1371/journal.pgen.1000774)
Supplement: Figure S2 — Alignment of the D. radiodurans ATCC13939 RecJ protein. Alignment of the D. radiodurans ATCC13939 RecJ protein with its TIGER sequence and the corresponding Deinococcus geothermalis, Termus thermophilus, and E. coli RecJ protein. The alignment was generated using Clone Manager program. Shading was based on amino acid identity (green boxes). (0.04 MB DOC) [file pgen.1000774.s002.doc]

*D.rad* ATCC13939 1 -----MSRP-AHWLLAPPASRDALLATMREWQVSPPVAQVLCGRDLRT--ELLALPLELTPNPAL---RE

*D. geothermalis* 1 MRRAAFSLPEARWLLAPPASRAALLESMRLWGVAPPLAQVLHARGLTP--AHLDAPLRLTPNPAL---RE

*T. thermophilus* 1 -----MRDR-VRWRVLPLPPLAQWREVMAALEVGPEAALAYWHRGFRRK-EDLDPPLALLPLKGL---RE

*E. coli* 1 ------MKQ-QIQLRRREVDETADLPA----ELPPLLRRLYASRGVRSAQELERSVKGMLPWQQLSGVEK

*D. rad*(TIGR seq) ----------------------------------------------------------------------

*D.rad* ATCC13939 60 AARHIVAAVREGKRIRIHGDYDADGVSATATLVLGLRAIG-ANVHGFIPHRLNEGYGIHPDRVPE-HAAA

*D. geothermalis* 66 AARRIVGAIRAGRRIRIHGDYDADGVSATATLILGLREVG-AEVHGFIPHRLNEGYGLHPDRVEE-HAAA

*T. thermophilus* 61 AAALLEEALRQGKRIRVHGDYDADGLTGTAILVRGLTALG-ADVHPFIPHRLEEGYGVLMERVPE-HLEA

*E. coli* 60 AVEILYNAFREGTRIIVVGDFDADGATSTALSVLAMRSLGCSNIDYLVPNRFEDGYGLSPEVVDQAHARG

*D. rad*(TIGR seq) 1 -----MAAVREGKRIRIHGDYDADGVSATATLVLGLRAIG-ANVHGFIPHRLNEGYGIHPDRVPE-HAAA

*D.rad* ATCC13939 128 ADLVVTVDCGVSNLDEVKSLLATGTEVVVTDHHAPGENFPEC-LVVHPHLTPDYDPDRHNLTGAGVAYHL

*D. geothermalis* 134 CDLLVTVDCGVTNLEEVRALLARGLDVIVTDHHAPGPDYPDC-LVVHPHRTTGYDPELHNLTGAGVAYHL

*T. thermophilus* 129 SDLFLTVDCGITNHAELRELLENGVEVIVTDHHTPGKTPPPG-LVVHPALTPDL---KEKPTGAGVAFLL

*E. coli* 130 AQLIVTVDNGISSHAGVEHARSLGIPVIVTDHHLPGDTLPAAEAIINPNLRDCNFPSK-SLAGVGVAFYL

*D. rad*(TIGR seq) 64 ADLVVTVDCGVSNLDEVKSLLATGTEVVVTDHHAPGENFPEC-LVVHPHLTPDYDPDRHNLTGAGVAYHL

*D.rad* ATCC13939 197 L-----------WAVYEELGRPEPRALLPLATLGTVADVAPLLGENRALVRAGLAEMARTEL-PGLRALM

*D. geothermalis* 203 L-----------WAVHEELGLPAPLELAGLATLGTIADVAPLIGENRALVRAGLDALATSTL-PGVRALL

*T. thermophilus* 195 L-----------WALHERLGLPPPLEYADLAAVGTIADVAPLWGWNRALVKEGLARIPASSW-VGLR-LL

*E. coli* 199 MLALRTFLRDQGWFDERNIAIPNLAELLDLVALGTVADVVPLDANNRILTWQGMSRIRAGKCRPGIKALL

*D. rad*(TIGR seq) 133 L-----------WAVYEELGRPEPRALLPLATLGTVADVAPLLGENRALVRAGLAEMARTEL-PGLRALM

*D.rad* ATCC13939 255 N--EKRVRQPTARDVAFILAPRINAAGRMGEADRALELLTTPSDHEAKSLAAYLEIRNQERRKIQDDMFA

*D. geothermalis* 261 N--AKRVERPSVRDVAFLLAPLVNAAGRLGEADLALQLLTTTSDHEARTLATYLESRNQERRVLQDRMYE

*T. thermophilus* 252 A--EAVGYTGKAAEVAFRIAPRINAASRLGEAEKALRLLLTEDAAEAQALVGELHRLNARRQTLEEAMLR

*E. coli* 269 EVANRDAQKLAASDLGFALGPRLNAAGRLDDMSVGVALLLCDNIGEARVLANELDALNQTRKEIEQGMQI

*D. rad*(TIGR seq) 191 N--EKRVRQPTARDVAFILAPRINAAGRMGEADRALELLTTPSDHEAKSLAAYLEIRNQERRKIQDDMFA

*D.rad* ATCC13939 323 QALQLADPNDPA-------LVLTHDDWHAGVMGIVASKLVETFNRPVYIVAQG-----KGSVRSTPGISA

*D. geothermalis* 329 EALGLADPEEPA-------IVVTKPDWHAGVMGIVASKLVETFHKPVYIVAQG-----KGSVRSTPGISA

*T. thermophilus* 320 KLLPQADPEAKA-------IVLLDPEGHPGVMGIVASRILEATLRPVFLVAQG-----KGTVRSLAPISA

*E. coli* 339 EALTLCEKLERSRDTLPGGLAMYHPEWHQGVVGILASRIKERFHRPVIAFAPAGDGTLKGSGRSIQGLHM

*D. rad*(TIGR seq) 259 QALQLADPNDPA-------LVLTHDDWHAGVMGIVASKLVETFNRPVYIVAQG-----KGSVRSTPGISA

*D.rad* ATCC13939 381 VQGLRESRDL----LGRFGGHPGAAGFSLDPQNFGALRERIHGYVRQFPTPV---PAVRLDAPLPVAALT

*D. geothermalis* 387 VEGLRYSHDL----LKRYGGHPGAAGFALDEANFEALRDRLHTYVRQFPRPV---PMWRLDAPLPTRAAT

*T. thermophilus* 378 VEALRSAEDL----LLRYGGHKEAAGFAMDEALFPAFKARVEAYAARFPDPV---REVALLDLLPEPGLL

*E. coli* 409 RDALERLDTLYPGMMLKFGGHAMAAGLSLEEDKFKLFQQRFGELVTEWLDPSLLQGEVVSDGPLSPAEMT

*D. rad*(TIGR seq) 317 VQGLRESRDL----LGRFGGHPGAAGFSLDPQNFGALRERIHGYVRQFPTPV---PAVRLDAPLPVAALT

*D.rad* ATCC13939 444 PELLSELSILEPFGEGNPRPLWHLRGPLTDTRLVGKQGDVLQFRFGGVKGMKYSERDDAAG-ERDVAAEL

*D. geothermalis* 450 PELVRQAAAFEPYGTGHPPPLWHMREVLSSTRLVGKRGDSLQFQIGGLRGIQHGERDAVPG-ERDLATHL

*T. thermophilus* 441 PQVFRELALLEPYGEGNPEPLFLLFGAPEEARRLG-EGRHLAFRLKGVRVLAWKQGDLALPPEVEVAGLL

*E. coli* 479 MEVAQLLRDAGPWGQMFPEPLFDGHFRLLQQRLVG-----------------------------------

*D. rad*(TIGR seq) 380 PELLSELSILEPFGEGNPRPLWHLRGPLTDTRLVGKQGDVLQFRFGGVKGMKYSERDDAAG-ERDVAAEL

*D.rad* ATCC13939 513 ALNEWKGRTSLELHAAALRPLAPLALAGTEEGL-PTLPRLNPREAMTFLKTGAAAYAEQGVATYLRDNVP

*D. geothermalis* 519 VRGEWRGQTRLELHGQALRPPARLSLDSPYTDA-PPLPRLDPKEAMNHLRAGASAYADGPVATYLREQVP

*T. thermophilus* 510 SENAWNGHLAYEVQAVDLR--KPEALEG---GIAPFAYPLPLLEALARARLGEGVYVPE-------DNPE

*E. coli*  514 -----------ERHLKVM--VEPV----------------------------------------------

*D. rad*(TIGR seq) 449 ALNEWKGRTSLELHAAALRPLAPLALAGTEEGL-PTLPRLNPREAMTFLKTGAAAYAEQGVATYLRDNVP

*D.rad* ATCC13939 582 GLTLLDTNAPHPGGDLILYGLPPESA-LRRWLHEAQEQGGRVAFALGPKTLAELDAALTLAKLLPDSHTE

*D. geothermalis* 588 GLTLLSPGAPHPGGELILYALPPEAD-LTRWLGE-----GRVAFAFGPKTLAELEGSLAHHPSL----TG

*T. thermophilus* 568 GLDY----ARKAGFRL----LPPEEAGLWLGLPPRPVLGRRVEVALGREARARLSAP-------PVLHTP

*E. coli* 525 ------------GGGPLLDG---------------------IAF--------NVDTA-----LWPDNGVR

*D. rad*(TIGR seq) 518 GLTLLDTNAPHPGGDLILYGLPPESA-LRRWLHEAQEQGGRVAFALGPKTLAELDAALTLAKLLPDSHTE

*D.rad* ATCC13939 651 AAQEAAADAYRSWQWAHHYR---VLNDAGWSASVYAMLGLPVPAALPKAAE---ALALAAG---------

*D. geothermalis* 648 AELEEAADAYRRWQWAHLYR---VLDDAGWNAAVRHLLGL-VQAPPCDARE---AALTAADD--------

*T. thermophilus* 623 EARLKAL-VHRRLLFAYERRHPGLFSEA--LLAYWEVNRVQEPAGSP-----------------------

*E. coli* 549 EVQLAYKLDINEFRGNRSLQ---IIIDNIW----------PI----------------------------

*D. rad*(TIGR seq) 587 AAQEAAADAYRSWQWAHHYR---VLNDAGWSASVYAMLGLPVPAALPKRRKRWRSLRVKASVRLPAARST

*D.rad* ATCC13939 -------------------------------

*D. geothermalis* -------------------------------

*T. thermophilus* -------------------------------

*E. coli* -------------------------------

*D. rad*(TIGR seq) 654 PGRFRVRLPAVRGRGSAAPVPLPYRAREWFH

**Figure S2**
