# Supplementary material for: A Major Role of the RecFOR Pathway in DNA Double-Strand-Break Repair through ESDSA in Deinococcus radiodurans
Source: PLoS Genet. 2010 Jan 15;6(1):e1000774. doi: 10.1371/journal.pgen.1000774 (PMC2806897; doi:10.1371/journal.pgen.1000774)
Supplement: Table S1 — Overview of primers used for construction of mutant strains, cloning, and diagnostic PCR experiments. (0.04 MB DOC) [file pgen.1000774.s004.doc]

**Table S1.** Overview of primers used for construction of mutant strains, cloning and diagnostic pcr experiments.

### Prime Primer sequence (5’ 3’) ^a^ Use

***recF* deletion**

PS313 ATT**GGATCC**TTGTAGGGGCGGCGCGGACC Amplification of *recF* upstream region (c)

PS314  CACGGCGCTGAACACTGCAC Amplification of *recF* upstream region

PS315 ATC**TCTAGA**CAGCGTGCCGCAGGCCATCG Amplification of *recF* downstream region

PS316 ACCTGCGGGTCGCTCATCTC Amplification of *recF* downstream region (c)

***recO* deletion**

EB73 ACGACCACGCGGTACATCTG Amplification of *recO* upstream region

EB74 AAT**GGATCC**TGACGATGCCGCTGCGGTTG Amplification of *recO* upstream region (c)

EB75 ATA**TCTAGA**GCGGCAACTCGTCCCAAGCG Amplification of *recO* downstream region

EB76 CGTTGCTGACGCCTCAGAAG Amplification of *recO* downstream region (c)

***recR* deletion**

EB69: TAGCGGCTTGTCCTCCAACG Amplification of *recR* upstream region

EB70: ATT**GGATCC**TGACAGCTCGCGGATGAGGG Amplification of *recR* upstream region (c)

EB71: ATA**TCTAGA**GCAACGTCCCGGTGACGAAG Amplification of *recR* downstream region

EB72: CGGCTGTCCACCTTGAACTG Amplification of *recR* downstream region (c)

***recA* deletion**

EB87Bis AATGGATCCGGCCTTGCTGCGTTCCTTGG Amplification of *recA* upstream region (c)

EB88Bis ACGGGTGGAAGACCTCAAGC Amplification of *recA* upstream region

EB89Bis TAA**TCTAGA**GGCAACGCGGGCGAAGCACC Amplification of *recA* downstream region

EB90Bis CGGCATCGGCATCAATGTGG Amplification of *recA* downstream region (c)

***uvrD* deletion**

EB97 AAT**GGATCC**TTGAGGGCTTGGAGCAGGTC Amplification of *uvrD* upstream region (c)

EB98 AGGCGTCAGCAGTAGGGAAG Amplification of *uvrD* upstream region

EB99 AAT**TCTAGA**GCTGTAGCCCGACGCTGTGG Amplification of *uvrD* downstream region

EB100 GGGCCTGAAATTCAGTGTCC Amplification of *uvrD* downstream region (c)

***recQ* deletion**

PS321 ATA**GGATCC**AGGATAGCGCGTTTACGTTG Amplification of *recQ* upstream region (c)

PS322 GCGTGCCGTCGTGGAACTTG Amplification of *recQ* upstream region

PS323 ATC**TCTAGA**TAAGGGCCGCCGGGCCACTC Amplification of *recQ* downstream region

PS324 GGGTCACGTCGAGGTGAAAC Amplification of *recQ* downstream region (c)

***recJ* deletion**

EB77 GCTGGGCAGTATCGCTGTTC Amplification of *recJ* upstream region

EB78 ATA**GGATCC**AGAGTCGCAGTCGCACTCAC Amplification of *recJ* upstream region (c)

EB79 ATA**TCTAGA**CGCCTTCCTGCTGCACGTTC Amplification of *recJ* downstream region

EB80 CGCGTAACCGACCTGATGAC Amplification of *recJ* downstream region (c)

**Amplification cassette**

PS177 ATT**GGATCC**TATACGGAACCTATACGGG Amplification of a *cat* cassette

PS178 ATT**TCTAGA**CGCGGCCGCACTTATTCA Amplification of a *cat* cassette (c)

Kan5Bam GGAA**GGATCC**GCATTCTGCCTCCAGCATCTC Amplification of a *kan* cassette

Kan3Xba GGAA**TCTAGA**GCAAGCAGCAGATTACG Amplification of a *kan* cassette (c)

EB87 AAT**TCTAGA**GATCCGTGTTTCAGTTAGCC Amplification of a *hph* cassette (c)

EB88 ATT**GGATCC**CGACGGCCAGTGAATTCGAG Amplification of a *hph* cassette

**Diagnostic primers**

***recF* deletion**

PS317 GAATGCCGTTGGGCATGAAC Verification of left junction *recF* deletion

PS318 TCGAGCGTCAGCACGCAGTC Verification of right junction *recF* deletion (c)

PS343 TCACGCCTTCCGGGAAATTC Verification of *recF* deletion (c)

***recO* deletion**

EB83 ATATGGTCGGCCAGCGCTTC Verification of left junction *recO* deletion

EB84 TTTGTGTTGGCCTTCCTCAG Verification of right junction *recO* deletion (c)

***recR* deletion**

EB81 TACTCGCCGTTGCGTTTGCC Verification of left junction *recR* deletion

EB82 GTGGAAACCGCAGCCAAAGC Verification of right junction *recR* deletion (c)

***recA* deletion**

EB91 TTCTCCTCGAAGCCGACCTG Verification of right junction *recA* deletion (c)

EB92 CAGATGCACGTCACCCTCTC Verification of left junction *recA* deletion

***recQ* deletion**

PS325 ACCAGCGAGTCAGGTTGAAG Verification of left junction *recQ* deletion

PS326 TGCGCATTTCGCCGTAGAGC Verification of right junction *recQ* deletion (c)

***recJ* deletion**

EB85 TTCTTCTGCTGGCGCTGCTC Verification of left junction *recJ* deletion

EB86 GTCCGAGGAACTGCTCTTTG Verification of right junction *recJ* deletion (c)

***uvrD* deletion**

EB101 CTCCCACTTGCCGACCAATC Verification of left junction *uvrD* deletion

EB102 TCTGACAGCCGCGCCTTAAC Verification of right junction *uvrD* deletion (c)

***cat* cassette**

PS93 TATCCAGCTGAACGGTCTGGTTA Verification of *cat* insertion, test for left junction (c)

PS94 TTAAACGTGGCCAATATGGACAACT Verification of *cat* insertion, test for right junction

***kan* cassette**

PS191 CTGGAGGTCGTGACTGTGAT Verification of *kan* insertion, test for left junction (c)

PS192 TTGCCATCCTATGGAACTGC Verification of *kan* insertion, test for right junction

***hph* cassette**

EB89 CGCCGATAGTGGAAACCGAC Verification of *hph* insertion, test for right junction

EB90 AGCTCGAATTCGATATCAAG Verification of *hph* insertion, test for left junction (c)

**Cloning**

***recF cloning***

PS410 ATAG**ACTAGT**ATGGCCGTGGCGGTGATGTC Amplification of *recF* (cloning in p11520)

PS411 ATCA**GGATCC**CATGTCGAGCGGGCCACTGC Amplification of *recF* (cloning in p11520) (c)

***recO* cloning**

PS402 ATT**GAGCTC**TACGACTCGGGAAACAGTTC Amplification of *recO* (cloning in p11520)

PS403 ATT**GGATCC**CTGGGTAGAGGGTCAAACAG Amplification of *recO* (cloning in p11520) (c)

***recR cloning***

PS414 CATT**GAGCTC**GGGCACGGCAAGGTGACCAG Amplification of *recR* (cloning in p11520)

PS415 ATTC**GGATCC**TGAGGGCAGCATGGCTCAGG Amplification of *recR* (cloning in p11520) (c)

***recJ* cloning**

PS441 GACTCCG**CATATG**AGCCGGCCTGCCCACTG Amplification of *recJ* (cloning in p13840)

PS442 ATTC**CTCGAG**TGCAGCAGGAAGGCGAACTG Amplification of *recJ* (cloning in p13840) (c)

^a^ Tags with restriction site are in bold. (c) Sequence is on the complementary strand
